# Comprehensive clinical evaluation of CAR-T cell immunotherapy for solid tumors: a path moving forward or a dead end?

**DOI:** 10.1007/s00432-022-04547-4

**Published:** 2022-12-24

**Authors:** Konstantinos Drougkas, Konstantinos Karampinos, Ioannis Karavolias, Ioannis-Alexios Koumprentziotis, Ioanna Ploumaki, Efthymios Triantafyllou, Ioannis Trontzas, Elias Kotteas

**Affiliations:** 1 Oncology Unit, Sotiria General Hospital, National and Kapodistrian University of Athens, 152 Mesogeion Avenue, 11527 Athens, Greece; 2 Department of Medicine, School of Medicine, National and Kapodistrian University of Athens, Athens, Attica Greece; 3 Department of Pathology, Yale University School of Medicine, New Haven, USA CT

**Keywords:** CAR T cells, Solid tumors, Immunotherapy, Chimeric antigen receptor, Tumor microenvironment

## Abstract

**Introduction:**

Chimeric Antigen Receptor (CAR)-T cell therapy is a form of adoptive cell therapy that has demonstrated tremendous results in the treatment of hematopoietic malignancies, leading to the US Food and Drug Administration (FDA) approval of four CD19-targeted CAR-T cell products. With the unprecedented success of CAR-T cell therapy in hematological malignancies, hundreds of preclinical studies and clinical trials are currently undergoing to explore the translation of this treatment to solid tumors. However, the clinical experience in non-hematologic malignancies has been less encouraging, with only a few patients achieving complete responses. Tumor-associated antigen heterogeneity, inefficient CAR-T cell trafficking and the immunosuppressive tumor microenvironment are considered as the most pivotal roadblocks in solid tumor CAR-T cell therapy.

**Materials and methods:**

We reviewed the relevant literature/clinical trials for CAR-T cell immunotherapy for solid tumors from Pubmed and ClinicalTrials.gov.

**Conclusion:**

Herein, we provide an update on solid tumor CAR-T cell clinical trials, focusing on the studies with published results. We further discuss some of the key hurdles that CAR-T cell therapy is encountering for solid tumor treatment as well as the strategies that are exploited to overcome these obstacles.

## Background

### CAR-T cell structure, molecular mechanism and generations

Chimeric Antigen Receptor (CAR)-T cell therapy is one of the most rapidly evolving approach in the field of immunotherapy, named adoptive cell therapy (ACT). In 2017, US Food and Drug Administration (FDA) approved the use of CD19- targeted CAR-T (Tisagenlecleucel, tisa-cel) for the treatment of young adults with refractory or relapsed (r/r) B-cell acute lymphoblastic leukemia (B-ALL) and of adults with r/r diffuse large B-cell lymphoma (DLBCL) (Brentjens et al. 2013; Davila et al. 2014; Grupp et al. 2013). However, advances of CAR-T therapy are lagging in solid tumors so far, rendering their application in the management of solid malignancies challenging (Haslauer et al. 2021).

**Fig. 1:**
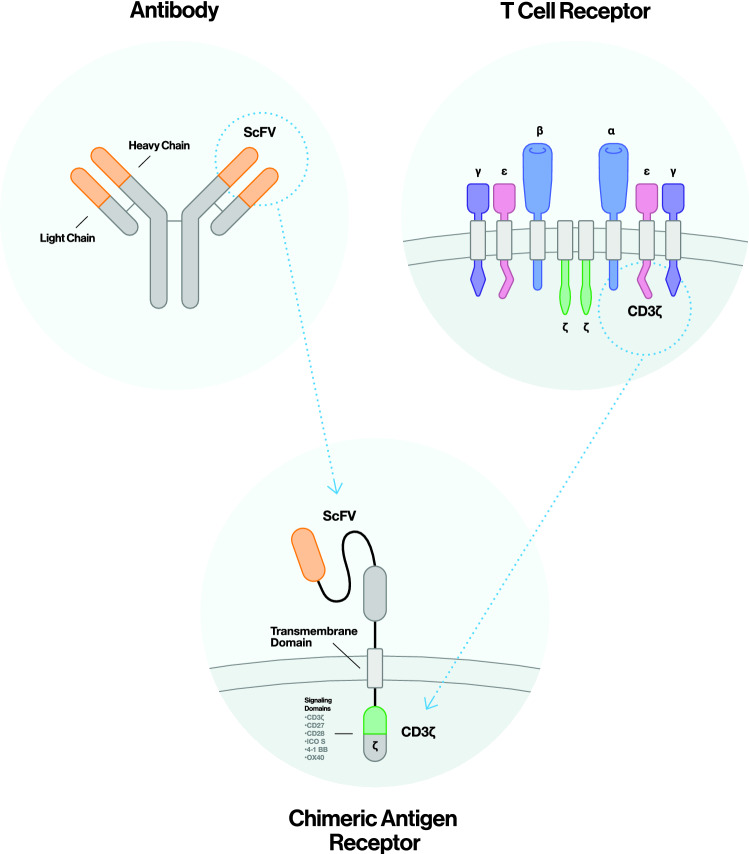
Schematic illustration of a CAR structure. A CAR is typically composed of a specificity-conferring scFv extracellular binding domain that is linked, via spacer/hinge and transmembrane domains, to a CD3ζ intracellular signaling moiety (less commonly an FcεRIγ domain) that can include one or more intracellular costimulatory domains (CD8, CD27, CD28, CD134, CD137, 41-BB, OX40). CARs can recognize target antigens in a non-MHC dependent manner. *CAR* chimeric antigen receptor, *ScFV* single-chain variable fragment

CAR-T cells originate from T cells which have integrated special transgenes into their genome, after a viral or nonviral- mediated gene transfer/ transduction process and clonal expansion. In detail, transferred genes are those that code for CARs, which are recombinant immunoglobulin T-cell receptors (TCRs), able to bind to cell surface antigens (Dotti et al. 2009). CARs are composed of a single-chain variable fragment (scFv) extracellular binding domain—containing variable fragment regions of antibodies – and a CD3ζ intracellular signaling moiety (less commonly an FcεRIγ domain), as well as some additional intracellular costimulatory domains, such as, but not limited to CD8, CD27, CD28, CD134, CD137, 41-BB, OX40 (Fig. 1) (Fesnak et al. 2016).

In contrast with TCR, found normally in native T cells, which exert their effects through major histocompatibility complex (MHC) -mediated binding process via antigen presenting cells (APCs), CARs are chimeric receptors, whose binding properties are not MHC/APCs restricted, thus being able to recognize and bind to any surface antigen for which they are specific (Aghajanian et al. 2022). Therefore, CARs are effective targeting tools even for tumor cells, with downregulated MHC expression or dysfunctional antigen processing, properties attributing TCR- related immune evasion. Nonetheless, unlike TCR’s expanded antigen binding range (both surface and intracellular proteins recognition), CARs activity remains limited to extracellular surface antigen recognition (Hanahan 2022).

Regarding CAR-T cells mechanism of action, their activation initiates upon binding of the extracellular scFv CAR portion to a specific tumor tissue antigen, named tumor-associated antigen (TAA). Therefore, upon binding, the intracellular moiety contributes to signal binding transduction, resulting in altered genomic expression through activation of several transcription factors, thus leading, among others, to cytokine production, granzyme and perforin release, death receptor ligand expression and overall CAR-T cell activation, survival, naive immune cell recruitment and antitumor cytotoxic activity (Larson et al. 2021). Apart from those effects, CAR-T cell activation contributes to immune memory formation of the recruiting cells, namely: Regulatory T-cells (Tregs), dendritic cells, natural killer (NK) cells, CD4 + /CD8 + T cells, macrophages (Dotti et al. 2009; Fesnak et al. 2016).

**Fig. 2:**
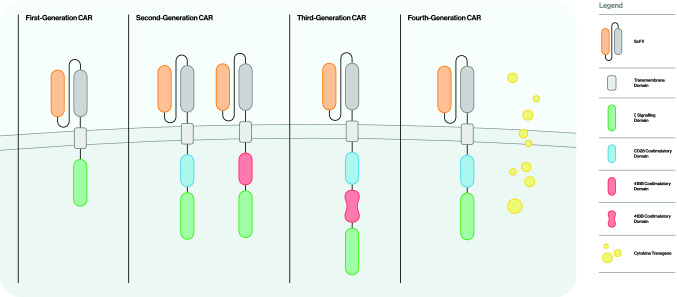
Schematic representation of the four generations of CARs. The first-generation CAR contains one intracellular signaling domain, most commonly the signaling domain of the CD3 TCRζ chain. A second intracellular co-stimulatory domain, typically consisting of either a CD28 or 4-1BB domain, was added in the second-generation CARs, enhancing CAR-T cell activation and proliferation. Third- generation CARs utilize dual co-stimulatory intracellular signaling, classically consisting of either a CD28 or a 4-1BB domain followed by either a CD28, a 4-1BB or an OX40 domain. Fourth-generation CARs co-express, on top of a second-generation CAR construct, an antitumor cytokine gene (i.e., IL-12) or costimulatory ligands (i.e., 4-1BBL). *CAR* chimeric antigen receptor, *ScFV* single-chain variable fragment, *TCR* T cell receptor, IL-12 interleukin-12, 4-1BBL 4-1BB ligand

CAR-T cell design has been comprised of four generations, with increasing target specifity and CAR-T activation effectiveness (Fig. 2). Kuwana et al., followed by Eshhar et al., introduced the first-generation CAR-T cells, reporting the first designed CAR-T cell, with an extracellular domain composed of an scFv portion, combined with an intracellular CD3ζ costimulatory domain (Eshhar et al. 1993; Kuwana et al. 1987). However, CD3ζ-CARs have been shown to elicit a moderate response, in terms of cytokine production and target CAR-T cell activation (Brocker and Karjalainen 1995).

Second-generation CAR-T cells emerged as a CAR-T cell conformation, with enhanced T-cell response, due to a single additional costimulatory intracellular molecule, added in the cytoplasmic moiety of CARs. Namely, some of the most common used molecules are CD28, 4-1BB, DAP10, OX-40 or ICOS, of which special attention should be drawn to CD28, a costimulatory molecule widely used to enhance cytokine production (Interleukin-2; ΙL-2) and overall, CAR-modified T cells expansion and persistence (Journal of Broker 1995; Maher et al. 2002; Savoldo et al. 2011; Kofler et al. 2011).

As a means of further enhancing signal transducing capacity and stimulation targeted effect, CARs got enriched with an additional costimulatory molecule, apart solely from CD28, resulting in a chimeric receptor composed of the following domains: scFV, CD28 or other, OX-40 or 4-1BB, CD3ζ (third-generation CAR-T cells) (Koehler et al. 2007). Hence, in various preclinical models, it was shown that a dual- costimulatory containing CAR is far more efficient than a single- costimulatory CAR, in terms of T-cell signaling strength and persistence (Hombach et al. 2012; Brentjens et al. 2007; Milone et al. 2009).

Recently, a next generation, hence 4^th^, CAR-T cells have been introduced, which have emerged as a means of rendering CAR- modified T cells resistant to cancer immune evasive activity. TRUCKs (T-cells redirected for universal cytokine killing) are CAR-T cells, which, on top of their CAR receptor, they coexpress an antitumor cytokine gene, predominantly interleukin-12 (IL-12) (Kowolik et al. 2006). Many cancer cells own a distinct phenotypic expression, which alters their stroma properties, thus creating the so-called tumor microenvironment (TME). The cytokine tumor profile attracts many inhibitory native immune cells, which protect tumor from immune recognition. Thus, TRUCKs confront this immunosuppressive tumor property, by expressing immune activating cytokines (i.e., IL-12), thus inducing native immune cell response towards cancer cells which have escaped from CAR-redirected T cells (antigen heterogeneity) and altering microenvironment immune-suppressive cells phenotype (Chmielewski and Abken 2015). More precisely, IL-12 has been shown to have several effects on cancer stroma, namely extracellular matrix alterations (reduced matrix metalloproteinases (MMPs), vascular endothelial growth factor (VEGF), induction of type 1 T helper (Th1) polarisation, NK cells activation, negative effect in angiogenesis and endothelial adhesion molecules (Chmielewski and Abken 2012; Hsieh et al. 1993; Hung et al. 1998; Curtsinger et al. 2003).

### CAR-T cell therapy in hematological malignancies

Following the first FDA approval in 2017, six CAR-T cell products have become available to date: Tisagenlecleucel (tisa-cel, Novartis: r/r B-ALL in young adults up to age 25, r/r DLBCL in adults), axicabtagene ciloleucel (axi-cel, Kite Pharma: r/r DLBCL, r/r primary mediastinal large B-cell lymphoma (PMBCL), r/r high grade B-cell lymphoma, and r/r DLBCL arising from follicular lymphoma (FL)), brexucabtagene autoleucel (brex-cel, Kite Pharma: r/r mantle cell lymphoma, r/r B-cell precursor ALL in adults), lisocabtagene maraleucel (liso-cel, Juno Therapeutics & Bristol Myers Squibb: r/r DLBCL, r/r high-grade B-cell lymphoma, r/r PMBCL, r/r FL grade 3B), idecabtagene vicleucel (ide-cel, Bristol Myers Squibb: r/r MM) and most recently ciltacabtagene autoleucel (cilta-cel, J&J, Legend biotech: r/r MM) (Kaliński et al. 1999). The first four CAR-T cell products are directed against CD19 while the latest, namely ide-cel and cilta-cel target B cell maturation antigen (BCMA). Notably, cilta-cel utilizes a dual epitope-binding nanobody-modulated CAR directed against 2 distinct BCMA epitopes (Chung et al. 2021). With regard to the costimulatory intracellular domain, Kite Pharma’s axi-cel and brex-cel utilize CD28 while the rest CAR-T cell designs contain 4-1BB.

Tisagenlecleucel was initially approved for B-ALL patients at 25 years of age or younger. This approval followed crucial studies that reported encouraging results with complete response (CR) rates ranging between 60 and 90% in patients with heavily pretreated, r/r B-ALL. In the next year, 2018, tisa-cel was additionally approved for the treatment of adults with r/r DLBCL, following promising data reported from a phase II clinical trial with an overall response rate (ORR) of 81% and relapse-free survival of 66% at 18 months (Zhao et al. 2018).

Around the same time, axicabtagene ciloleucel was also approved for r/r large B cell lymphoma and DLBCL. The most significant study for this CAR-T cell product was a phase I/II clinical trial demonstrating an ORR of 83% and CR in approximately half of patients with r/r DLBCL with some remissions persisting for over two years (Schuster et al. 2019; Neelapu et al. 2017).

Next, in 2020, brexucabtagene autoleucel was approved for the treatment of r/r mantle cell lymphoma. In a multicenter, phase 2 trial brex-cel showed an ORR of 85% and a CR of 59% at six months, while progression-free survival (PFS) and overall survival (OS) were 61% and 83%, respectively at 12 months (Locke et al. 2019).

Subsequently, in 2021, lisocabtagene maraleucel was approved for r/r adult DLBCL high-grade B-cell lymphoma, PMBCL and FL grade 3B. The most pivotal study regarding liso-cel, was TRANSCEND NHL 001, a phase I open label trial, in which an ORR of 73% and a CR of 53% were achieved, while PFS and OS were 51% and 75% respectively after 6 months (Wang et al. 2020).

Regarding MM, the second most frequently diagnosed hematologic malignancy and the most common type of myeloma (Abramson et al. 2020**),** two CAR-T cell therapies have been approved as of May 2022 for its r/r forms, with several others being currently investigated. First, idecabtagene vicleucel (March 2021), which in the phase 2 KarMMa trial exhibited an ORR of 73% and a CR of 33% with PFS and OS being 8.8 and 24.8 months respectively (Kumar et al. 2017). Second, ciltacabtagene autoleucel (February 2022), that in the CARTITUDE-1 phase 1b/2 trial demonstrated outstanding results: The ORR was 97%, the stringent CR was 67% and the 12-month progression- free rate was 77% (Munshi et al. 2021).

Despite major breakthroughs in hematological neoplasms, CAR-T cell therapy against solid tumors remains in an infant stage. The most important emerging question that needs to be addressed in the near future is to what extent can CAR-T cell therapy prove beneficial for patients with solid tumors when compared to patients dealing with hematological malignancies. In this review we aim to provide an update of the available evidence from clinical trials of CAR-T cell therapy in solid malignancies and to discuss challenges and future perspectives.

## Clinical investigation of CAR-T cell therapy in solid tumors

**Fig.3:**
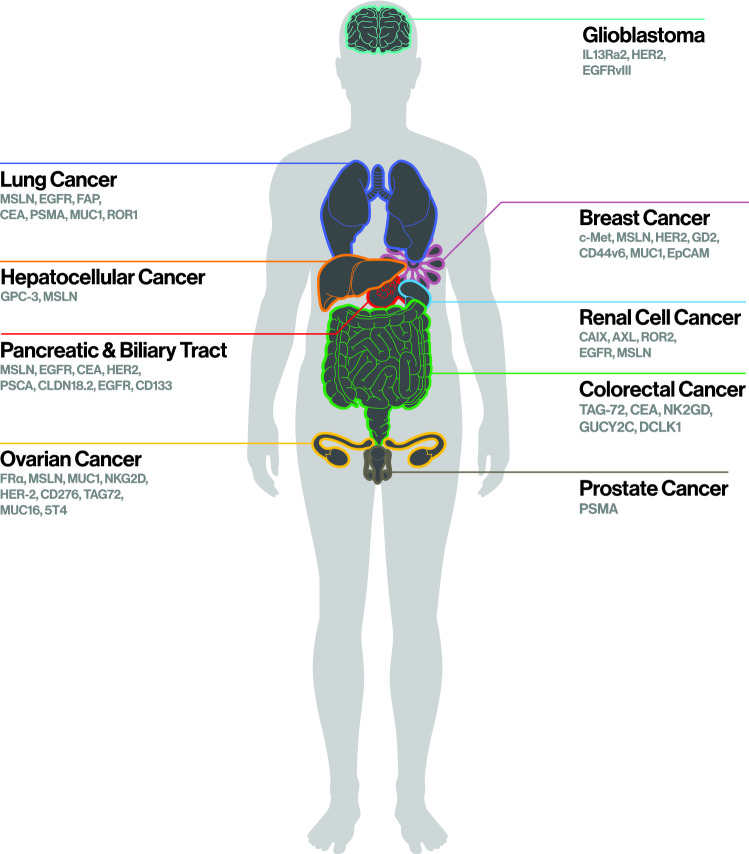
Graphical representation of tumor-associated antigens that are currently being utilized in CAR-T cell therapy for a variety of solid tumors. CAR chimeric antigen receptor, CEA carcinoembryonic antigen, MSLN mesothelin, *EGFR* epidermal growth factor receptor, *EGFRvIII* epidermal growth factor receptor variant III, *HER-2* human epidermal growth factor receptor-2, *GPC-3* glypican-3, *PSMA* prostate-specific membrane antigen, CLDN18.2 claudin, *IL13RA2* interleukin 13 receptor alpha 2, *PSCA* prostate stem cell antigen, *TAG-72* tumor-associated glycoprotein-72, *FAP* fibroblast activating protein, *CAIX* carbonic anhydrase IX, *c-Met* c-mesenchymal- epithelial transition factor, *FR-α* folate receptor alpha, *GD2* disialoganglioside, *MUC1* mucin-1, MUC16 mucin-16, *NKG2D* natural killer group 2d, *EpCAM* epithelial cell adhesion molecule, *ROR* receptor tyrosine kinase-like orphan receptor, *GUCY2C* guanylyl cyclase 2C, *DCLK1* doublecortin-like kinase 1, *AXL AXL* receptor tyrosine kinase

The combination of the previous success in hematological malignancies, in which CAR-T cell therapy has documented high rates of long-lasting and persistent disease remission and the novel breakthroughs in preclinical models for solid tumors, has resulted in more than 250 clinical trials utilizing CAR-T cells against solid tumors being conducted by a great number of medical centers internationally. In this part, we evaluate clinical trials with published results (Table 1) focusing on CAR- T cell target antigen, dosage, safety and efficacy while we additionally mention novel targets that exhibit promising features in preclinical studies and are currently explored in several ongoing clinical trials (Fig. 3).

**Table 1:** CAR-T cell clinical trials for solid tumors with published results

Cancer	Antigen	Identifier	Patients	Dosage–Route of administration	Persistence	Outcome	Adverse events	Notes	Reference
Pancreatic–biliary tract	EGFR	NCT01869166	16	3.48 × 10^6^/kg (range 1.31 to 8.9 × 10^6^/kg); Intravenous	Baseline levels within 1 month	PR: 4 patients (2–4 months) SD: 8 patients (2–4 months) PD: 2 patients NE: 2 patients	Grade ≥ 3 fever/fatigue, nausea/vomiting, mucosal/cutaneous toxicities, pleural effusion and pulmonary interstitial exudation (on-target/off-tumor toxicity); reversible	Positive correlation between CAR-T cell persistence and number of Tcm	Katz et al. 2020a)
	MSLN	NCT02159716	15 (n = 5, PDAC)	1–3 × 10^7^ or 1–3 × 10^8^/m2; Intravenous	28 days	SD: 11/15 patients (28 days); 3/8 patients (follow-up, 2–3 months) PFS: 2.1 months	Low-grade fatigue, nausea, vomiting, confusion, diarrhea, fever; 1 patient with grade 4 sepsis	Antibodies against anti-MSLN CAR-T cells	Feng et al. 2017)
	MSLN	NCT01897415	6	3 times weekly for 3 weeks; Intravenous	Transient (mRNA CAR-T cells)	SD: 2 patients (3.8 and 5.4 months respectively)	Grade ≥ 3 back pain (1) and abdominal pain (1)	Construction utilizing mRNA electroporation	Pang et al. 2021)
	HER-2	NCT01935843	11	2.1 × 10^6^/kg (range 1.4–3.8 × 10^6^/kg); Intravenous	Up to 80 days (9/11 patient’s serum CAR transgene copy numbers were > twofold of the baseline value at first evaluation timepoint)	PR: 1 patient (4.5 months) SD: 5 patients (4.8 months)	Mild-to-moderate nausea/vomiting, fatigue, myalgia/arthralgia, lymphopenia; Grade 3 acute fever/chill (1) and abnormal transaminase elevation (1)		Haas et al. 2019)
	PSCA	NCT02744287	9	3 + 3 cell dose escalation, 1.25 × 10^6^–2.5 × 10^6^/kg; Intravenous	Up to > 3 weeks	SD: 4 patients (≥ 8 weeks) 2 minor responses (not confirmed) PD: 2 patients	Most common AEs were fatigue and nausea	Utilization of Rim-inducible costimulatory domain (molecular safety switch)	Beatty et al. 2018; Becerra et al. 2019a)
	CLDN18.2	NCT03159819	12 (n = 5, PDAC)	1–5 cycles, total of 0.5–55 X 10^8^ cells; Intravenous	NA	CR: 1 patient PR: 3 patients SD: 5 patients PD: 2 patients PFS: 130 days	No grade 4 AEs except for lymphopenia, neutropenia; All CRS observed were grade 1 or 2		Berdeja et al. 2021)
	EGFR and CD133	NCT01869166 NCT02541370	1	EGFR: 2.2 × 10^6^/kg, 2.1 × 10^6^/kg (first and second cycle, respectively) CD133: 1.22 × 10^6^/kg	EGFR: Baseline levels within 2 months CD133: Baseline levels within 4.5 months	EGFR: PR (8.5 months) CD133: PR (4.5 months)	EGFR: Mild chills, fever, fatigue, vomiting, muscle soreness, 9-day lasting lower fever and a grade 2 skin rash CD133: Fever, chills fatigue, intermittent upper abdominal dull pain,, sporadic pinpoint hemorrhages and Grade 3 congestive skin rash	Successive administration of two different CAR-T cell products	Liu et al. 2020)
	CEA	NCT02850536	1	4 infusions of 1 × 10^10^ cells; Hepatic artery infusions	NA	Complete metabolic response of metastatic disease (13 months)	Grade 1–3 fever, chills/rigors, tachycardia, hypotension, diarrhea, fatigue, mild abdominal distension, myalgias,,thrombocytopenia, electrolyte dysfunction and transient elevations in liver function tests		Becerra et al. 2019b)
Metastatic colorectal	TAG-72	C-9701 C-9702	10 (C-9701) 6 (C-9702)	1 × 10^8^ – 1 × 10^10^ cells; C-9701: Intravenous C-9702: Hepatic artery infusions	C-9701: Up to 14 weeks C-9702: Up to 48 weeks	Best response was PD	C-9701: Chills, fever, dizziness, paresthesia, headache, tachycardia, myalgia, hypoxia, grade-3 chills (1), retinal artery occlusion (1) C-9702: Fever, abdominal pain, increased bilirubin, headache, nausea, vomiting, anemia, transient; Hypotension and mild congestive heart failure (1)	First human clinical trials of CAR-T cells for solid tumors; IFN-α co-administration; Antibodies against TAG-72 binding domain	Zhang et al. 2017)
	CEA	NCT01373047	6	1 × 10^8^ –1 × 10^10^ cells; Hepatic artery infusions	NA	SD: 1 patient (23 months) PD: 4 patients NE: 1 patient	All patients experienced transient grade 1–2 liver enzyme elevations; Grade 3 fever and tachycardia (1), attributed to IL-2 co-administration; Grade 3 liver enzyme elevations (1)		Thistlethwaite et al. 2017)
	CEA	NCT02416466	6	3 infusions of 1 × 10^10^ cells; Hepatic artery infusions	NA	SD: 3 patients mOS: 6.9 months (range 3.8–10.8 months)	Grade 1–2 liver function test elevations, fever, hypereosinophilia, and edema; Grade 3 colitis (2), fever (2), and edema (2); Hypertensive crisis (1)	Tumoricidal synergism of SIR -Sphere brachytherapy and anti-CEA CAR-T cells	Hege et al. 2017)
	CEA	NCT01212887	14	1 × 10^9^–5 × 10^10^ cells; Intravenous	Up to 2 weeks	SD: 7 patients (3,7 months; range 2,7–5,1 months) PD: 7 patients	Acute respiratory toxicity (pulmonary infiltrates, respiratory distress) in Cohort 4 patients (4). (premature termination of the trial)	On-target/off-tumor toxicity	Katz et al. 2020b)
	CEA	NCT02349724	10	1 × 10^5^–1 × 10^8^/kg; Intravenous	Up to 4–6 weeks	SD: 7 patients PD: 2 patients NE: 1 patient	Only grade 2 fever (2) was related to CAR-T cell therapy; 1 instance of duodenal perforation was attributed to dyspepsia	3rd generation CAR-T cells	Sureban et al. 2019)
Hepatocellular carcinoma	CD133	NCT02541370	21	0.5 × 10^6^—2 × 10^6^/kg; Intravenous	Up to 12 months	PR: 1 patient SD: 14 patients PD: 6 patients PFS: 6.8 months (range 4.3–8.4 months) mOS: 12 months (range 9.3–15.3 months)	Nausea, anemia, thrombocytopenia, constipation, hypotension, bilirubinemia; Grade 3 anemia (2), hyperbilirubinemia (4)	Phase II trial; Association between plasma biomarkers and outcome (PFS, OS)	Carl et a. 2018)
	GPC3, MSLN	NCT03198546	6 (n = 4, HCC)	0.25 × 10^6^–8.7 × 10^6^/kg; Intravenous, Intratumor, hepatic artery infusions, intraperitoneal	NA	CR: 1 patient (9 months) PR: 1 patient SD: 2 patients (6 months; range 3–9 months) PD: 2 patients	Fever, fatigue	Car-T cells engineered to secrete IL-17 and CCL19	Zhan et al. 2019)
Lung (malignant pleural mesothelioma)	MSLN	NCT01355965	2 (MPM, PDAC)	2 × 10^8^ – 1 × 10^9^ cells; Intravenous, intratumor, intraperitoneal	Transient (mRNA CAR-T cells)	PR: 1 patient (6 month) SD: 1 patient	Anaphylactic reaction with subsequent grade 4 cardiac arrest, respiratory failure, DIC and CRS (1); Grade 4 jejunal obstruction, grade 3 abdominal pain, and grade 2 lymphcytosis (1)	Severe anaphylactic reaction due to formation of IgE antibodies against the murine- based SS1 scFv CAR portion	Schuberth et al. 2013; Petrausch et al. 2012)
	MSLN	NCT02414269	31	1 × 10^5^–6 × 10^7^/kg; Intrapleural (intracavitary and intratumoral)	NA	Complete metabolic response: 2 patients PR: 5 patients SD: 4 patients	Grade 1–2 toxicities; Grade 3 CRS (1) and dyspnea (1)	Infusions under image guidance by computed tomography or ultrasound	Carpenito et al. 2009; Zhang et al. 2021)
	EGFR	NCT03182816	9	1 × 10^6^–3 × 10^6^/kg; Intravenous	Up to 28 days	PR: 1 patient (> 13 months) SD: 6 patients PD: 2 patients PFS: 7.13 months (range 2.71–17.10 months) mOS: 15.63 months (8.82–22.03 months)	Grade 1–2 fever, chills, muscle weakness, nausea/vomiting, skin rash; Grade 3 fever (1)	piggyBac transposon system	Zhao et al. 2010)
	FAP	NCT01722149	4	1 × 10^6^/kg Intrapleural	CAR-T cells detected in the blood of 1 patient	NE	Grade 2 upper respiratory infection; Grade 3 lymphopenia (1), thromboembolic event (2) (not related to CAR-T cell therapy)		Ghosn et al. 2022; Adusumilli et al. 2019)
Renal cell carcinoma	CAIX	DDHK9729/P00.0040C	12	2 × 10^7^–2 × 10^9^ cells; Intravenous	Up to 4 weeks	NE	Grade 3–4 liver enzyme disturbances	CAIX expression on bile duct epithelium (on-target/off-tumor toxicity)	Beatty et al. 2014)
Breast	c-MET	NCT01837602	6	3 × 10^7^–3 × 10^8^ cells; Intratumor	Transient (mRNA CAR-T cells)	SD: 1 patient PD: 2 patients Death: 3 patients	Grade I erythema occurred at the intratumoral injection sites, myalgia/arthralgia		Teachey et al. 2016)
	c-MET	NCT03060356	7 (n = 4, BC)	1 × 10^8^; Intravenous	Transient (mRNA CAR-T cells)	SD:4 patients PD:3 patients	Grade 1–2 anemia, fatigue, malaise		Khoury et al. 2001)
Ovarian	FR-a	NCT00019136	14	3 × 10^9^–5 × 10^10^ cells; Intravenous	Up to 12 months (1); most up to 3 weeks	PD: 14 patients	Grade 1–2 fatigue, nausea, vomiting, edema, diarrhea, bilirubinemia, leukopenia, thrombocytopenia; Grade 3–4 hypotension, dyspnea, leukopenia, rigors, sinus tachycardia, diarrhea (attributed to IL-2 administration)	Development of an inhibitory factor against CAR-T cells	Tchou et al. 2017)
Prostate	PSMA	BB-1ND12084	5	1 × 10^9^–1 × 10^10^ cells; Intravenous	Up to 4 weeks	PR: 3 patients NE: 2 patients	Grade 1–2 fatigue, intermittent low-grade fevers, myalgias (due to IL-2 administration) Grade 3–4 neutropenia (5), neutropenic fever (5), thrombocytopenia (3), anemia (1), hypocalcemia (1), hypophosphatemia (1), appendicitis (1)	Inverse correlation between IL-2 levels and CAR-T cell engrafment	Slovin et al. 2022)
	PSMA	NCT01140373	7	1 × 10^7^ to 3 × 10^7^/kg; Intravenous	Up to 2 weeks	SD: 2 patients (6–16 months) PD: 2 patients NE: 3 patients	All patients had intermittent fever spikes (up to 39 °C)		Arcangeli et al. 2020)
	PSMA	NCT03089203	13	1 × 10^7^–3 × 10^8^/kg; Intravenous	NA	3 patients with PSA reduction of ≥ 30%; 1 patient with PSA reduction of ≥ 98%;	Grade ≥ 2 CRS (5), including one patients (≥ 98 PSA reduction) with grade 4 CRS, concurrent sepsis and death	Dominant-negative TFG-β receptor CAR-T cells	Chekmasova et al. 2010)
	PSMA	NCT04249947	10	0.25 × 10^6^–15 × 10^6^/kg; Intravenous	NA	3 patients with PSA reduction of ≥ 50%; 1 patient with PSA reduction of ≥ 99%;	Grade 3 cytopenia (6), infection (1); Grade 1–2 CRS (5); Grade ≥ 3 CRS with macrophage activation syndrome/uveitis (1)	iCasp9-based safety switch	Owens et al. 2018)
Glioblastoma	IL13Ra2	NCT00730613	3	1 × 10^7^–1 × 10^8^/kg (3 infusions of 1 × 10^7^, 5 × 10^7^, 1 × 10^8^/kg followed by 9 doses of 1 × 10^8^/kg; Intracavitary via a catheter/reservoir system; Intratumor	Up to 14 weeks	PR: 2 patients (12 months; 10–14 months) PD: 1 patient	Grade 3 headache (2), grade 3 neurologic event (shuffling gait and tongue deviation) (1)		Morgan et al. 2012)
	IL13Ra2	NCT02208362	1	2 × 10^6^–10 × 10^10^; Intracavitary (6 infusions) Intraventricular (10 infusions)	Levels detectable up to 149 days	CR: 7.5 months	Grade 1–2 headaches, generalized fatigue, myalgia, olfactory auras		Vitanza et al. 2021)
	HER-2	NCT03500991	Estimated enrollment 45 patients (early interim analysis of 3 patients)	1 × 10^7^ –10 × 10^7^; (weekly infusions for 3 weeks followed by a week off) Intracavitary, intraventricular	NA	Evidence of local CNS immune activation in all patients (CXCL10 and CCL2 increase in CSF)	Mild grade 1–2 toxicities; Grade 3 headache (2), grade 3 back pain (1)		Humphrey et al. 1990)
	HER-2	NCT01109095	17	1 × 10^7^–1 × 10^8^/m2; Intravenous	Up to 12 weeks	PR:1 patient (8 months) SD: 7 patients (> 6 weeks) PD: 8 patients	NA		Brown et al. 2016)
	EGFRvIII	NCT02209376	10	1.75 × 10^8^–5 × 10^8^ cells; Intravenous	Up to 1 month	SD: 1 patient (> 18 months) PD: 2 patients NE: 7 patients	Mainly grade 1–2 nervous system toxicities; Grade 3 left ventricular systolic dysfunction (1), muscle weakness (1), facial muscle weakness (1), headache (1), intracranial hemorrhage (1), seizure (2); Grade 4 cerebral edema (2)		Brown et al. 2015)

*CR* complete response, *PR* partial response, *SD* stable disease, *PD* progressive disease, *NE* not evaluable, *NA* not available, *PFS* progress free survival, *mOS* median overall survival, *AEs* adverse events, CRS cytokine release syndrome, Tcm central memory T- cells, Rim rimiducid, *IFN-α* interferon-alpha, *IL-2* interleukin-2, *IL-17* interleukin-17, *TGF-β* transforming growth factor beta, *SIR* selective internal radiation, *CCL2* chemokine ligand 2, *CCL19* chemokine ligand 19,* CXCL10* CXC motif chemokine ligand 10, *iCasp9* inducible caspase 9, *CAR* chimeric antigen receptor, *CEA* carcinoembryonic antigen, *MSLN* mesothelin, *EGFR* epidermal growth factor receptor, *EGFRvIII* epidermal growth factor receptor variant III, *HER-2* human epidermal growth factor receptor-2, *GPC3* glypican-3, *PSMA* prostate-specific membrane antigen, CLDN18.2 claudin, IL13RA2 interleukin 13 receptor alpha 2, *PSCA* prostate stem cell antigen, *TAG-72* tumor-associated glycoprotein-72, *FAP* fibroblast activating protein, *CAIX* carbonic anhydrase IX, *c-MET* c-mesenchymal- epithelial transition factor, *FR-α* folate receptor alpha

### Pancreatic and biliary tract cancer

Currently, in clinical trials with published results the most frequently exploited target antigens are mesothelin (MSLN), epidermal growth factor receptor (EGFR), carcinoembryonic antigen (CEA), human epidermal growth factor receptor-2 (HER2), prostate stem cell antigen (PSCA), claudin 18.2 (CLDN18.2, NCT03159819 (Berdeja et al. 2021)) and C133. The majority of these trials utilize a second-generation CAR construct. N. Pang et al. designed a 3rd/ 4th generation of anti-MSLN CAR-T cells (NCT03198546), engineered to secrete IL-17 and chemokine ligand 19 (CCL19) (Zhan et al. 2019). Notably, L. Beatty et al. generated anti-MSLN CAR-T cells, after genetic modification via mRNA electroporation to transiently express a CAR specific for MSLN, as a means of preventing unexpected adverse events (AEs) (Pang et al. 2021). In another phase I clinical trial (NCT02744287), BPX-601 CAR-T cells, a T-cell product engineered to contain a PSCA-CD3ζ CAR was generated in combination with the small molecule rimiducid (Rim)-inducible MyD88/CD40 costimulatory domain, which serves as a molecular “switch” for T-cell activation and proliferation (Beatty et al. 2018; Becerra et al. 2019a).

A dose escalating, multiple injections pattern is the most commonly used strategy regarding CAR-T cell dosage, with a starting point ranging from 1.25 × 10^6^ and 1 × 10^10^ (fixed dose), after a preparatory lymphodepleting regimen, usually including cyclophosphamide. Other chemotherapy options were nab-paclitaxel and fludarabine. To enhance CAR-T cell trafficking and tumor penetration, percutaneous hepatic artery infusions (HAI) were performed in a number of clinical trials (NCT03198546, NCT02850536). Notably, S. Katz et al., in HITM- SURE clinical trial (Becerra et al. 2019b), along with 3 HAIs of 1 × 1010 cells at weekly intervals, also administered continuous intravenous (IV) infusions of IL-2, similarly to NCT01373047 clinical trial, to support in vivo CAR-T cell persistence. In another clinical trial (NCT01869166) patients additionally received palliative radiotherapy as a means to improve tumor-associated pain (Katz et al. 2020a). Lastly**,** in one patient with advanced unresectable/metastatic cholangiocarcinoma (CCA) resistant to radiotherapy and chemotherapy, two different CAR-T cell products, namely anti-EGFR and anti- CD133, were successively administered. This case suggested that repeated infusions of CAR-T cells, as well as successive administration of two different CAR-T cell products, following resistance or relapse to the first target, can potentially prolong CAR-T cell persistence in vivo and PFS (Liu et al. 2020). In the same case, following promising preclinical findings that programmed cell death protein 1 (PD-1) blockade enhances CAR-T cell function, 2 cycles of anti-PD-1 monoclonal antibody were also administered; however, not showcasing significant improvement.

In most clinical trials, a robust peripheral engraftment and expansion of CAR-T cells was observed, whereas CAR-T cell persistence was relatively modest with CAR transgene copy numbers most commonly declining to baseline levels within 1–2 months. Y.Liu et colleagues made a unique, worth-mentioning finding, positively correlating CAR-T cell persistence with the number of central memory T-cells (Tcm) in infused anti-EGFR CAR-T cells (NCT01869166). Besides CAR-T cell persistence, the enrichment of Tcm in infused CAR-T cells was positively related with clinical response (Katz et al. 2020a). Another study provided new insights into CAR-T cell expansion and persistence in vivo and the impact of lymphodepletion on these features. Specifically, anti-MSLN CAR-T cells showed modest expansion and poor persistence and their levels were almost undetectable by day 28 after infusion. Lymphodepleting regimen did improve the initial expansion of CAR-T cells (near-10- fold increase) but had no effect on persistence. Additionaly, in the same study, out of 14 patients evaluated, 10 of them developed antibodies against anti-MSLN CAR- T cells, leading to the hypothesis that anti-CAR antibodies and subsequent immune-mediated elimination of CAR-T cells was a contributing factor to poor persistence; however, there was no correlative evidence that these antibodies directly affected CAR-T cell persistence (Feng et al. 2017).

Regarding clinical responses, stable disease (SD) and partial response (PR) were the most common best overall responses with the median PFS typically ranging from 4 to 5 months. Additionally, in a non- negligible number of cases, disease progression was observed. Nevertheless, CRs were not absent. A patient with advanced pancreatic carcinoma that progressed with local lymph node metastasis (sized 24 × 33 mm in positron emission tomography/computerised tomography (PET- CT) scan) revealed CR via CT staging with the affected lymph node now measuring 8.3 × 9.6 mm after 5 infusions of anti-MSLN CAR-T cells (NCT03198546) (Zhan et al. 2019). Similarly, after 3 HAIs of CEA- specific CAR-T cells, complete metabolic response was established in a patient with poorly differentiated pancreatic adenocarcinoma and synchronous liver metastases, after constant evaluation with sequential PET-CT imaging (NCT02850536) (Becerra et al. 2019b).

Regarding overall toxicities and AEs, no case of cytokine release syndrome (CRS) or neurotoxicity AEs was detected. The most common AEs were reversible, low- grade AEs, namely fever, fatigue, nausea, vomiting and febrile episodes. Unique AEs included grade≥3, cutaneous/ mucosal toxicities (NCT01869166), neutropenia and lymphocytopenia (grade 4 AEs). However, typically, hematological toxicities, while present, were most probably related to the conditioning regimen. Additionally, post-infusion AEs including upper gastrointestinal hemorrhage, C-reactive protein (CRP) and IL-6 increase were also observed (NCT01935843) (Haas et al. 2019). Notably, in a specific clinical trial (NCT01869166), on-target/off-tumor toxicity occurred with anti-EGFR CAR-T cell therapies, as EGFR is present in most normal epithelial cells. Characteristically, two of the treated patients developed clinically manageable pleural effusion and pulmonary interstitial exudation toxicities (Katz et al. 2020a).

### Colorectal cancer

To date, clinical trials with published results in colorectal cancer (CRC) mainly utilize CEA as the TAA in CAR-T cell engineering. In C-9701 and C-9702, the first human clinical trials of CAR-T cells for solid tumors and specifically CRC, the exploited target-antigen was tumor-associated glycoprotein-72 (TAG-72). Most recently, antigens including natural killer group 2d (NK2GD) and guanylyl cyclase 2C (GUCY2C) are some of the most prominent novel targets based on preclinical studies (Feng et al. 2018; Deng et al. 2019), and several clinical trials exploiting these TAAs are currently ongoing. Furthermore, according to recent evidence, doublecortin-like kinase 1 (DCLK1)-targeted CAR-T cells effectively eradicate primary and metastatic colon cancer cells, rendering DCLK1 a promising target for future endeavors (Magee et al. 2018).

In the majority of clinical trials with published results, researchers utilize first or second-generation CAR constructs. Notably, Zhang et al., in a trial with enrolled CRC patients (NCT02349724), suggested that third generation of CAR with CD28 and CD137 signaling did not exhibit enhanced cytokine excretion and cytotoxicity profile than second generation with CD28 signaling (Sureban et al. 2019).

The most frequently tested CAR-T cell dosage scheme is centered on IV or successive dose-escalating HAIs, with a starting point ranging from 1 × 10^5^ to − 1 × 10^10^ (fixed dose) and an ending point of 5 × 10^10^. Concurrently, in most clinical trials, preconditioning regimens including cyclophosphamide and/ or fludarabine, are co-administered. In two clinical trials (NCT01373047, NCT01212887), patients also received IL-2 systemic infusions, as a means of promoting CAR-T cells peripheral blood expansion. Moreover, based on the previously established ability of interferons to upregulate TAG-72 expression, in C-9701 and C-9702 trials, researchers co- administered interferon-alpha (IFN-α), along with each dose of anti-TAG-72 CAR-T cells. Unfortunately, the data that emerged from these two trials were insufficient to demonstrate whether IFN-α co-administration led to significant TAG-72 expression upregulation to overcome the immune-evading mechanism of antigen-loss escape (Zhang et al. 2017). Remarkably, in HITM-SIR (NCT02416466) phase 1b clinical trial, researchers decided to explore the safety and potential tumoricidal synergism of Selective Internal Radiation (SIR)-Sphere brachytherapy following CEA- specific CAR-T HAI, acknowledging the established synergistic interaction between immunotherapy and radiation. Specifically, radiation therapy, through tumor lysis and antigen release, may promote the immune system to enable CAR-T cells or form a favorable cytokine milieu (Hege et al. 2017). However, further studies are required to confirm the potential additive or synergistic role of SIR to CAR-T cell therapy.

In most clinical trials, relatively poor CAR-T cell engraftment and persistence was observed. Specifically, peripheral persistence was mostly short- termed, with CAR-T cell levels declining to baseline after a few weeks. More specifically, in C-9701 and C-9702 trials**,** rapid CAR-T cells clearance was partly attributed to the immunogenicity of anti-TAG-72 CAR-T cells resulting in the induction of interfering antibodies against the TAG-72 binding domain (Zhang et al. 2017). Thistlethwaite et al. indicated that high intensity pre-conditioning chemotherapy led to elevated levels of systemic IFNγ and IL-6, suggesting successful CAR-T cell immune activation in vivo and therefore, significantly higher anti-CEA CAR-T cell engraftment levels (Katz et al. 2020b). Interestingly, Zhang et al. inferred that the CAR-T cells second infusion could achieve similar levels of CAR-T cells expansion and persistence as the first administration. The same researchers tested Flow Cytometry (FCM) for CAR-T cells detection in peripheral blood, using a novel reagent (Protein L) or a recombinant CEA protein with His tag. However, CAR-T cells in peripheral blood were detected only by a sensitive method using polymerase chain reaction (PCR), but not by FCM (Sureban et al. 2019).

In the majority of clinical trials, no remarkable clinical responses were documented, while the best overall response was stable disease. Katz et al. estimated neutrophil:lymphocyte ratios (NLR) changes as a measure of early CAR-T clinical response, since high NLR is associated with poor prognosis. It was shown that patients with serologic responses, that is, a decreasing trend of serum CEA antigen, were more likely to present a statistically significant NLR fold- change (Thistlethwaite et al. 2017).

Concerning overall safety, CAR-T cell therapy for CRC was well tolerated, with typically limited serious AEs. The most common AEs were manageable, low- grade AEs, namely fever, fatigue and mild liver enzyme elevations. Remarkably, patients in HITM and HITM-SIR, despite receiving a very high intratumoral dose of CAR-T cells in the liver, showed no evidence of severe hepatic or biliary toxicity (Hege et al. 2017). Unique AEs included hypereosinophilia, edema, colitis, hypertensive crisis (NCT02416466) and duodenal perforation (NCT02349724). A case of grade 3 fever and tachycardia (NCT01373047) was attributed to systemic IL-2 administration. Moreover, in the same trial, instances of platelet count decrease, alopecia and gastritis were related to lymphodepletion. Distinctively, in C-9701 and C-9702, researchers observed low-grade CRS events, most prominently in patients with high CAR-T cell engraftment levels. Last, in one clinical trial the development of on-target off-tumor toxicity, clinically evident as transient, acute respiratory toxicity in patients (CEA expression in lung epithelium), resulted in the premature termination of the trial (NCT01212887) (Katz et al. 2020b).

### Hepatocellular carcinoma

Based on encouraging preclinical results regarding 7 × 19 CAR-T cells (CAR-T cells concomitantly secreting IL-7 and CCL19) (Katz et al. 2015; Adachi et al. 2018; June et al. 2018), this phase I clinical trial (NCT03198546) was designed to evaluate safety, tolerability and clinical activity of IV/intratumoral/intrahepatic artery injections of glypican-3 (GPC3)- / MSLN- specific 7 × 19 CAR-T cells in 6 patients with immunohistochemically confirmed GPC3- or MSLN- positive advanced hepatocellular carcinoma (HCC), pancreatic cancer, or ovarian cancer (OC), following lymphodepleting chemotherapy (Zhan et al. 2019). No grade 2–4 AEs, CRS, neurotoxicity or treatment- related death was documented. One pancreatic cancer patient achieved CR, one HCC patient achieved PR and two other HCC patients reached SD. The above results demonstrate the reasonable therapeutic potential of 7 × 19 CAR-T cell therapy for advanced solid tumors with GPC3/MSLN expression. Despite disease burden and active metastatic sites, some patients reached SD state, with negligible treatment- related AEs.

In this single-center, single-arm, open-label, phase I/II clinical trial (NCT02541370), adults with histologically confirmed and measurable advanced HCC received CD133-directed CAR-T cells (CART-133) infusions, using a standard 3 + 3 dose escalation approach. Primary endpoints of the study included safety and tolerability in phase I and PFS and OS in phase II (Carl et al 2018). Considering safety, most of the documented AEs were of grade 2, while no CRS or neurologic toxicity events were documented. Of 21 evaluable patients, 1 presented a PR, 14 had SD for 2 to 16.3 months, and 6 had PD after CAR-T cell infusion, while the median OS was 12 months and the median PFS was 6.8 months. However, 4 patients with pre-existing obstructive lesions experienced hyperbilirubinemia (grade 3 toxicity). Notably, therapy outcome was correlated with the baseline levels of several proangiogenic and inflammatory factors: VEGF, soluble VEGF receptor 2 (sVEGFR2), stromal cell-derived factor (SDF)-1, and endothelial progenitor cells (EPC) counts, while also, an association between changes of these biomarkers’ levels after CART-133 infusion with survival was observed. In conclusion, in patients with advanced HCC, CART-133 cell therapy demonstrates manageable safety profile and promising antitumor activity.

### Lung cancer

Until recently, CAR- T cell clinical trials with published results in patients with lung cancer have been focusing on malignant pleural mesothelioma (MPM), using mainly MSLN as the target antigen. However, fibroblast activating protein (FAP) has also been tested as a potential TAA in patients with MPM, while EGFR has been exploited in patients with non- small cell lung cancer. In most clinical trials, the specific generation of CAR-T cells is not announced, while three of them utilize second generation CAR constructs (NCT01355965, NCT01897415, NCT01722149). In two consecutive phase I clinical trials from the University of Pennsylvania (NCT01355965, NCT01897415), in an effort to reduce the associated safety concerns of viral vector gene transduction- based CAR-T cells engineering, anti-MSLN CAR-T cells were constructed using RNA electroporation (Huang et al. 2020; Dai et al. 2020). Similarly, Zhang et al. exploited the non-viral piggyBac transposon system to generate EGFR-specific CAR-T cells, due to the fact that, compared to viral systems, the piggyBac transposon system is a simpler, more economical, and alternative way to introduce CAR transgenes into T cells (Zhao et al. 2010). Lastly, Ghosn et al. engineered anti-MSLN CAR-T cells, incorporating inducible caspase-9 (iCasp9) safety switch, as a means of limiting on-target, off-tumor toxicities of CAR-T cell therapy (Carpenito et al. 2009; Zhang et al. 2021).

Regarding CAR-T cell dosage scheme, the most frequently used was a dose- escalating pattern, with a starting point ranging from 1 × 10^5^ to 2 × 10^8^ CAR- T cells were predominantly administered intravenously or directly intrapleurally. Notably, in a recent clinical trial (NCT02414269) with MPM patients, interventional radiologists administered the respective CAR-T cell product through intracavitary or intratumoral infusions under image guidance by computed tomography or ultrasound (Carpenito et al. 2009). In another clinical trial (NCT01722149), due to safety concerns, lowered doses of FAP-specific CAR-T cells (1 X 10^6^ cells/kg) were injected and despite this subtherapeutic dose, CAR-T cells expansion in systemic circulation was documented (Ghosn et al. 2022; Adusumilli et al. 2019).

To date, there is not enough evidence about CAR-T cell expansion and persistence in patients with MPM; however few clinical trials have made certain observations regarding these aspects. First, in the clinical trials utilizing RNA-electroporation for anti-MSLN CAR-T cell construction (NCT01355965, NCT01897415), poor CAR-T cell persistence with rapidly decreasing levels was in agreement with the biodegradable nature of the MSLN transgene (Schuberth et al. 2013; Petrausch et al. 2012). Nevertheless, the same researchers suggested that it is possible to administer multiple, more frequent injections of CAR-T cells that only temporarily express the selected transgenes, avoiding the accumulation of tolerized CAR-T cells and therefore enhancing both CAR-T cell persistence and tumor infiltration (Huang et al. 2020). Second, Zhang et al. attributed the lack of CAR-T cell expansion to several potential factors such as the low level of the antigen-bearing cells in the blood, the relatively low dose of CAR-T cells adopted in this trial (NCT03182816) (Zhao et al. 2010).

Regarding clinical activity, SD and PR were the most frequent best overall responses. In addition, disease progression was reported in a significant number of cases. Complete PET scan- based metabolic response was achieved in two patients receiving iCasp9 anti-MSLN CAR-T cells.

Concerning overall safety and tolerbality, CAR-T cell therapy for lung cancer was well-tolerated, showcasing a good safety profile in most instances, with grade 1 to grade 3 fever being the most frequent AE. Interestingly, Beatty et al. indicated that the mRNA electroporation design can reduce potential “on target/ off- tumor” toxicities due to the transient expression of the CAR in T cell surface (Huang et al. 2020). Lastly, there was a case of a severe anaphylactic reaction occurring within minutes after the third IV infusion of anti-MSLN CAR-T cells in a patient with MPM, that was attributed to the formation of IgE antibodies against the murine- based scFv CAR portion (Schuberth et al. 2013).

### Renal cell carcinoma

The only published clinical study of CAR-T cell therapy in patients with renal cell carcinoma (RCC), was a phase I/II trial conducted to assess the proof of concept and safety of using first generation CAR-T cells engineered to express a CAR for the TAA carboxy-anhydrase-IX (CAIX) for the treatment of CAIX + metastatic RCC (Beatty et al. 2014**).** Twelve patients were assigned in three cohorts and were treated with two cycles of multiple dose-escalating IV infusions of anti-CAIX CAR-T cells (2 × 10^7^–2 × 10^9^ cells/kg). Subcutaneous IL-2 was also administered as a means of enhancing CAR-T cell in vivo anti-tumor activity. Infused CAR-Ts were transiently detectable in the circulation, maintaining their antigen-specificity after post-treatment isolation. Although there were no clinical responses recorded, multiple recommendations for future trials have emerged from this study. First, patients developed antibodies and cellular immune responses against CAR-T cells, therefore highlighting the necessity to further examine the format and immunogenicity of CARs and specifically how the latter correlates with CAR-T cell persistence. Second, CAR-T cell infusions induced liver enzyme disturbances resulting in cessation of treatment in four out of eight patients. This was due to the development of on-target/ off-tumor toxicity as CAIX is expressed in the bile duct epithelium and CAR-T cells infiltrated around bile ducts.

Other clinical trials utilizing different potential target antigens including AXL receptor tyrosine kinase (AXL), receptor tyrosine kinase-like orphan receptor 2 (ROR2), EGFR and MSLN for RCC are currently ongoing. (NCT03393936, NCT03960060, NCT01869166, NCT03638206).

### Breast cancer

C-mesenchymal-epithelial transition factor (c-Met) is a well-known molecule which is overexpressed in breast tissue and breast cancer tissue, irrespectively of the variety of breast cancer subtypes, displaying several physiological functions. Thus, it has emerged as an important breast cancer TAA target (Beatty et al. 2014; Lamers et al. 2016). Most importantly, regardless of hormone receptor/HER2 expression profile, c-Met overexpression in breast cancer tissue has been well established (Ho-Yen et al. 2015; Teachey et al. 2016). Nevertheless, c-Met does not constitute a breast cancer tissue- specific antigen, since it is expressed at low levels on healthy tissues. To limit on-target off-tumor toxicity and to assess safety and feasibility of intratumoral injections of c-Met transfected- CAR-T cells, a phase I clinical trial in 6 metastatic breast cancer patients was conducted (NCT01837602). The first cohort of patients received a single intratumoral injection of c-Met CAR-T cells, at a dose level of 3 X 10^7^ cells, whereas the second cohort a higher dose of 3 X 10^8^ cells. Post-injection AEs were present; however they were deemed irrelevant of the treatment action, and thus the treatment was considered well-tolerated. Clinical responses were not observed, albeit that immunohistochemistry analysis of tumor specimens revealed extensive tumor necrosis, macrophage infiltration and c-Met loss of immunoreactivity, all of which could be indicative of CAR-T cells selective targeting and capacity of eliciting an inflammatory response within TME (Teachey et al. 2016).

In another phase I clinical trial (NCT03060356), IV administration of mRNA-electroporated c-Met-specific CAR-T cells in metastatic breast cancer or r/r melanoma patients was assessed in terms of safety, feasibility and tolerability. It was terminated due to funding reasons. Concerning AEs, no CRS or grade 3 toxicities were observed, while most common AEs were of grade 1 or 2, namely anemia, fatigue, and malaise, revealing a good overall safety profile. Concerning clinical responses, the best achieved response was stable SD (4/7 patients), while 50% (2/4) of breast cancer patients experienced disease progression (Khoury et al. 2001).

In addition, a recent phase I/II clinical trial (NCT04430595) has been designed to investigate the safety and feasibility of HER2-, disialoganglioside (GD2)-, CD44v6- specific CAR-T cells (4SCAR T cells) in subjects with breast cancer, as well as one more recent phase 1 trial (NCT04020575) has emphasized on using mucin (MUC)- targeting CAR-T cells (huMNC2-CAR44 cells) in advanced MUC1^+^ breast cancer. Patients with nasopharyngeal carcinoma or breast cancer have been enrolled in a phase I clinical trial, which investigated the safety and tolerability of epithelial cell adhesion molecule (EpCAM)- specific CAR-T cells, in terms of treatment-related AEs and maximum tolerated dose (MTD).

### Ovarian cancer

The first published clinical study of CAR-T cell therapy in patients with OC was a phase I clinical trial (NCT00019136) conducted to assess the safety of first-generation folate receptor alpha (FRα)-specific CAR-Τ cells (Tchou et al. 2017). In the first cohort, patients were treated with 3 treatment escalation dosages of FRα-specific CAR-T cells, in combination with high-dose IL-2. In the second cohort, patients were treated with 2 cycles of dual-specific CAR-T cells (reactive to both FRα and allogeneic cells) followed by subcutaneous immunization with allogeneic peripheral blood mononuclear cells (PBMCs) per cycle. Regarding treatment- related toxicities, serious AEs were attributed to high-dose IL-2 administration. There was no reduction in tumor burden as shown by a number of observations. First, tracking radiolabeled FRα-specific CAR-T cells exhibited lack of specific localization to tumor site except in one patient with detectable accumulation of CAR-T cells in a peritoneal metastasis. Second, CAR-T cells were present in the circulation in large numbers for only 2 days after administration, quickly declining and being barely detectable after 1 month in the majority of patients treated. Third, an inhibitory factor against the CAR-Ts was progressively developed in the serum of three patients, drastically limiting anti-tumor responses.

In an already mentioned clinical trial (NCT03198546), 1 patient with recurrent, refractory OC was treated with anti-MSLN CAR-T cells engineered to secrete IL-17 and CCL19 (Zhan et al. 2019). A total of two intra-abdominal infusions were administered with neither infusion-related nor therapy-related serious AEs. No tumor remission was observed and by day 38 of therapy, staging evaluation showed disease progression.

Other ongoing clinical trials utilize different potential antigen targets for OC such as MUC1, NKG2D, HER-2 and CD276 (NCT04025216, NCT03018405, NCT04511871, NCT04670068) while in vitro studies show promising findings exploiting novel tumor-specific antigens, namely TAG72, MUC16 and 5T4 (Shah et al. 2020; Kershaw et al. 2006; Murad et al. 2018). Lastly, some of the ongoing clinical trials explore the prospect of direct peritoneal administration to overcome the challenge of poor tumor trafficking. (NCT03585764, NCT02498912).

### Prostate cancer

Currently, all four clinical trials with published results for prostate cancer (PC) utilize prostate-specific membrane antigen (PSMA) as the target antigen. Remarkably, Narayan et al. developed the first-in-human clinical trial, in which PSMA-specific dominant-negative transforming growth factor β (TFG-β) receptor CAR-T cells were exploited (NCT03089203). Specifically, based on the ability of PC to secrete TGF-β for the inhibition of anti-tumor immunity, the investigators hypothesized that engineering anti-PSMA CAR-T cells insensitive to TGF-β (dominant negative TGF-β receptor) will enhance CAR-T cell ability to infiltrate, proliferate, and mediate antitumor responses in PC (Chekmasova et al. 2010). Moreover, Slovin et al. incorporated an iCasp9-based safety switch on their CAR construct (NCT04249947). In another recent clinical trial, the piggyBac transposon system was utilized to generate anti-PSMA CAR-T cells (Owens et al. 2018). This novel non- viral engineering system is believed to produce a high percentage of stem cell memory T cells, supporting in vivo CAR-T cells expansion and persistence (Narayan et al. 2022).

An IV dose- escalating, multiple injection pattern is the most commonly used dosage scheme, with a starting point ranging from 0.25 × 10^6^ to 1 × 10^9^, following a preconditioning lymphodepleting regimen consisting of cyclophosphamide and fludarabine. In one clinical trial (BB-1ND12084) low- dose IL-2 was co- administered, to sustain infused CAR-T cell activation. Interestingly, Junghans et al., described an unexpected inverse correlation between IL-2 levels and CAR-T cell engraftment, with administered IL-2 being depleted up to 20-fold with high engraments (Slovin et al. 2022). Compared to clinical trials on other solid tumors, CAR-T cells expansion and persistence did not significantly differ, with CAR-T cell levels declining after 3–4 weeks.

Concerning clinical activity, in the majority of clinical trials, clinical responses in patients treated with PSMA- specific CAR- T cells were evaluated considering prostate-specific antigen (PSA) serologic changes. More specifically, in a clinical trial, two-of-five patients achieved PSA responses with PSA declines of 50% and 70% and PSA delays of 78 and 150 days respectively (Slovin et al. 2022). Notably, in an ongoing phase I trial (NCT03089203), three patients demonstrated a PSA reduction of ≥ 30% with CAR-T cell suppression following upregulation of inhibitory soluble molecules in the TME, therefore highlighting the need to explore superior multipronged strategies against the TME in future studies (Chekmasova et al. 2010). Additionaly, early results of a recent clinical trial (NCT04249947) have demonstrated promising results regarding CAR-T cell antitumor responses. First, PSA-specific antitumor activity was documented in a total of 7 patients, with PSA declines of > 50% (*n* = 3) and > 99% (*n* = 1) noted. Second, three-of-four patients who underwent pre- and post-treament fluorodeoxyglucose (FDG) PSMA-PET imaging, exhibited significant to complete reduction of abnormal uptake in metastatic sites. Third, post-treatment tumor biopsy performed in one patient revealed infiltration and elimination of tumor cells by anti-PSMA CAR-T cells (Owens et al. 2018).

Concerning AEs and toxicities, in the two older clinical trials (NCT01140373 (Arcangeli et al. 2020), BB-1ND12084) none of the patients experienced any CAR-T cell treatment-related high- grade AEs, while the most common AE was intermittent febrile episodes. Paradoxically, in the most recent clinical trials (NCT03089203, NCT04249947), severe AEs were present. More precisely, Narayan et al. described AEs, referencing patient cases of grade ≥ 2 CRS, including one case of > 98% PSA reduction, grade 4 CRS and death due to concurrent sepsis. Furthermore, it was also mentioned that acute elevations in inflammatory cytokines were directly associated with manageable high-grade CRS episodes (Chekmasova et al. 2010). Similarly, in the most recent clinical trial (NCT04249947) CRS was observed in 6 patients, with one of them developing macrophage activation syndrome/uveitis (only grade ≥ 3 CRS event) while CRS marker elevations were moderate. In the same trial, other common side effects were cytopenias, infections and constitutional symptoms most probably due to lymphodepletion, while manageable ocular manifestations were reported in 3 patients (Owens et al. 2018).

### Glioblastoma

To date, interleukin 13 receptor alpha 2 (IL13Ra2) and HER2 are the most frequently tested TAAs in clinical trials with published results regarding CAR-T cell therapy in glioblastoma. Additionally, epidermal growth factor receptor variant III (EGFRvIII) originates from a novel tumor-specific gene rearrangement that codes for a unique protein expressed in approximately 30% of gliomas and is a promising target for CAR-T cell therapy (Junghans et al. 2016). Therefore, to the best of our knowledge there are two clinical trials with posted results that utilize this target antigen (NCT02209376, NCT01454596). Regarding the exploited CAR generation technology, in the majority of clinical trials, second generation CARs are being utilized. Interestingly, in one clinical trial (NCT01454596) the CAR construct containing the CD- 28 and 4-1BB co-signaling elements (third-generation CAR) was chosen by the researchers, based on previous evidence from animal model studies that the presence of additional signaling domains is associated with a better survival of CAR-T cells (Slovin et al. 2013).

In most clinical trials, CAR-T cells’ route of administration is intracavitary/ intracranial, via a catheter/reservoir system, while most commonly, multiple injections of the respective CAR-T cell product are infused. Specifically, in “IL13 zetakine” clinical trial (NCT00730613), the first in human pilot study, assessing safety and feasibility of CAR-T cells targeting IL13Ra2, a total of 12 escalating intracavitary doses (three initial infusions of 1 × 10^7^, 5 × 10^7^, 1 × 10^8^ cells/kg followed by 9 additional doses of 1 × 10^8^ cells/kg) were administered in two patients. In a follow-up trial (NCT02208362), one patient, with recurrent multifocal leptomeningeal glioblastoma involving both cerebral hemispheres received 6 intracavitary CAR-T cell infusions (an initial infusion of 2 × 10^6^ cells/kg followed by five infusions of 10 × 10^6^ cells/kg) through a catheter device. Remarkably, due to new emerging lesions and disease progression 10 additional intraventricular CAR-T cell treatment cycles were delivered via a second catheter device placed in the right lateral ventricle. Additionally, in a recently initiated clinical trial (NCT03500991), HER2-specific CAR-T cells were administered intracavitary or intraventricularly with a multiple locoregional injections pattern of a weekly dose of CAR-T cells for three weeks,

followed by a week off, an examination period, and then another course of weekly doses for three weeks (Humphrey et al. 1990). Lastly, it is worth mentioning that in two specific clinical trials, CAR-T cells were administered intravenously, following again a dose- escalating pattern (NCT02209376, NCT01109095).

Regarding clinical and antitumor activity, promising results have emerged from several clinical trials. First, in “IL13 zetakine” clinical trial patients developed temporary therapy-related brain inflammation and persistent necrosis at the tumor site following each infusion of anti-IL13Ra2 CAR-T cells (detected by increased MRI Gd-enhancement and increased signal on fluid attenuated inversion recovery (FLAIR) images). Remarkably, brain inflammation appeared to correlate directly with IL13Rα2 antigen expression, since it was most prominent in the two patients with the highest IL13Rα2 levels. Additionally, histopathological analysis of tumor tissue from one patient before and after CAR-T cell administration indicated decreased IL13Ra2 expression within the tumor, further enhancing evidence of anti-tumor activity of anti-IL13Ra2 CAR-T cells (Morgan et al. 2012). Furthermore, in the follow-up clinical trial (NCT02208362), a patient who received a total of 16 CAR-T cell regional infusions, demonstrated dramatical decrease (77% to 100% reduction) in all tumor sites (both intracranial and spinal) and the patient progressively returned to normal life activities, sustaining this clinical response for about 7.5 months (Vitanza et al. 2021). In another clinical trial utilizing anti-EGFRvIII CAR-T cells (NCT02209376 (Brown et al. 2015)) one patient experienced SD for over 18 months of follow-up while seven out of ten patients underwent post-therapy surgical resection, thus allowing pathologic study findings, which showed detectable CAR-T cell trafficking to active glioblastoma multiforme (GBM) sites, as well as EGFRvIII reduced expression in five out of seven patients, all indicating clinical activity of the study therapy. However, in situ evaluation of TME demonstrated increased and robust expression of inhibitory molecules after CART-EGFRvIII infusion, therefore highlighting the importance of overcoming immunosuppressive changes in TME to enhance the efficacy of EGFRvIII- directed strategies in GBM. Lastly results from a phase I trial exploiting anti-HER2 CAR-T cells revealed partial response in 1 patient, SD in 7 patients for 8 weeks to 29 months and progressive disease in 8 patients (16 evaluable patients) with researchers highlighting that evaluation of anti-HER2 CAR-T cells in GBM patients in a phase 2b study is warranted (Brown et al. 2016).

Concerning overall safety, CAR-T cell therapy for GMB was well tolerated, while all routes of administration (intracavitary, intraventricular and IV) indicated a good safety profile with typically limited serious AEs. Few instances of clinically manageable neurologic events, mostly headaches and seizures were also present. (NCT00730613, NCT02209376, NCT01109095).

### Recent progress

Very recently, presented at European Society for Medical Oncology (ESMO) Congress 2022, four early-phase studies, with two of them being strictly CAR- T cell- based (O’Rourke et al. 2017; Ahmed et al. 2017), highlighted the clinical potential of immunotherapy, as a reasonable treatment option in advanced solid tumors. Different technologies, with convincing efficacy and mixed tolerability, were explored; two studies utilized CAR-T-cell therapies (O’Rourke et al. 2017; Ahmed et al. 2017), one utilized a vaccine-targeted approach (Mackensen, et al. 2022) and one utilized a TCR T-cell therapy (Fang, et al. 2022).

More specifically, the first-in-human phase I clinical trial (NCT04503278) in patients with claudin 6 (CLDN6)- positive r/r solid tumors was designed to investigate safety and tolerability of an innovative, hybrid therapeutic approach, comprising two components: CLDN6- specific CAR-T cells and CLDN6-encoding CAR-T cell-Amplifying RNA Vaccine (CARVac), designed to expand adoptively transferred CAR-T cells and improve their persistence (O’Rourke et al. 2017). Following lymphodepletion, the bifurcated (monotherapy and combination) 3 + 3 design comprises CLDN6- specific CAR-T cells dose escalations for monotherapy and CAR-T cells dose escalations combined with CLDN6 CARVac, applied repeatedly after CAR-T cells infusion with an intra-patient dose escalation (25 up to 50 μg). Treatment- related AEs were attributed to lymphodepletion or asymptomatic transaminase/ lipase elevations. In particular, the following adverse events were observed: pancytopenia, hemophagocytic lymphohistiocytosis in the context of dose limiting toxicities (DLTs), as well as manageable CRS of grade 2 and 3. In terms of clinical response, out of 21 evaluable patients, 7 had PR, 6 had PD and most interestingly, 8 had SD, with 6 of them presenting with tumor shrinkage and 1 of them with post- 18 weeks negative PET-CT and negative serological tumour marker results.

Next, the first-in-human, open-label, multi-centers trial (NCT05028933) in patients with EpCAM- positive relapsed/ refractory gastrointestinal (GI) tumors was designed to examine the safety, efficacy and cytokinetic profile of IMC001, an EpCAM- specific CAR-T cell based immunotherapy. More precisely, patients were treated with a classic 3 + 3 design (0.3, 1 or 3 × 10^6^ cells/kg) with either separate IMC001 escalated doses (monotherapy) or IMC001 escalated doses combined with radiofrequency or microwave ablation (Ahmed et al. 2017). Regarding AEs, although no DLTs were observed, all patients developed more than grade 3 haematological toxicities, one patient developed autoimmune hepatitis, while two other patients developed grade 1–2 CRS. Preliminary efficacy results showed that 4 out of 5 evaluable patients showed SD and 1 patient, receiving the lowest treatment dosage, showed PD. In conclusion, IMC001 shows a manageable safety profile and reasonable anti-tumor activities at the initial dosage level in patients with refractory EpCAM + cancers of the GI system.

A recent update on safety and efficacy data of SURPASS (NCT04044859), the phase I clinical trial of ADP-A2M4CD8 in patients with antigen melanoma- associated antigen A4 (MAGE-A4)- positive unresectable or metastatic tumors, was presented at ESMO Congress 2022. ADP-A2M4CD8 is a next-generation, T-cell immunotherapy, based on the transduction of leukapheresis- obtained T-cells with a lentiviral vector carrying a T-cell receptor with enhanced affinity for a specific peptide and CD8α co-receptor genes. Currently updated data further support the favorable safety profile of ADP-A2M4CD8, while also providing encouraging evidence of clinical activity in patients with MAGE-A4- positive unresectable or metastatic tumors, especially gastroesophageal and ovarian tumors, for which also, two phase 2 trials are to be initiated (Fang et al. 2022).

## Overcoming challenges of CAR-T cell therapy in solid tumors

In this section we discuss the pivotal challenges associated with CAR-T cell therapy against solid tumors and the most prominent strategies that are currently developed to overcome them. Although detailed analysis of the obstacles in CAR-T cell therapy is beyond the scope of this review, the challenges listed below are the most significant barriers interfering with the effectiveness of CAR-T cell therapy and disturbing the desirable transition to everyday clinical practice (Table 2).

**Table 2:** CAR-T cell therapy main challenges and potential solutions

Main challenges of CAR-T cell therapy	Potential solutions
Choosing tumor-specific antigen	
Diverse expression of TAA in cancer cells Variable and changing levels of antigen expression in different tumor sites Presence of TAA in healthy tissues resulting in cross reactions with regional non-tumor cells	Co-administration of different CAR-T cell products Combining vectors for two separate CARs Bispecific CARs (Dual CAR, tanCAR, iCAR) Trivalent CAR-T cells TRUCKS-synNotch system Nanobody-based antigen recognition domain Targeting cancer stem cells
CAR-T cell trafficking and tumor penetration	
Tight connections with tumor-surrounding cells, presence of blood vessels, fibroblasts, and ECM proteins, signaling molecules and decreased levels of oxygen / nutrients Presence of dense fibrotic matrix in the tumor site Mismatching of endogenous T-cell chemokine receptors with tumor-secreted chemokines	Local administration of CAR-T cells in the tumor site Implantable biopolymer devices for direct delivery Transgenic expression of chemokine receptors on CAR-T cells Combination of CAR-T cells with oncolytic viruses Heparinase-secreting CAR-T cells FAP-specific CAR-T cells Co-administration of anti-VEGF antibodies
Immunosuppressive tumor microenvironment	
Presence of immune suppressor cells (Tregs, MDSCs, TAMs) Secretion of cytokines, growth factors and chemokines (IL-4, IL-10 and TGF-β) Presence of immune checkpoint molecules / inhibitory pathways (PD-1 or CTLA-4) Increased levels of adenosine and reactive oxygen species	Combination of CAR-T cells and immune checkpoint inhibitors Engineering PD-1 deficient CAR-T cells Depletion of Tregs and/or MDSCs CAR-T cells expressing dominant negative TGF-beta type II receptor Manufacturing CAR-T cells that secrete anti-cancer cytokines (IL-12, IL-15) TRUCKS-synNotch system

*CAR* chimeric antigen receptor, *TAA* tumor-associated antigen, tanCAR tandem CAR, iCAR inhibitory CAR, *TRUCKS* T cells redirected for antigen-unrestricted cytokine-initiated killing, synNotch synthetic notch, *FAP* fibroblast activation protein, *VEGF* vascular endothelial growth factor, Tregs regulatory T cells, *MDSCs* myeloid-derived suppressor cells, TAMs tumor-associated macrophages, *TGF-β* transforming growth factor-β, *PD-1 *programmed cell death protein 1, *CTLA-4* cytotoxic T-lymphocyte-associated protein 4, *IL* interleukin

### Choosing tumor-specific antigen

The diverse expression of TAAs in cancer cells is a major barrier to the effectiveness of CAR-T cell therapy against solid tumors. Unlike hematological malignancies where a TAA, such as CD19 in ALL, is uniformly expressed, most solid tumors do not express a single tumor specific antigen (Kyi et al. 2022). Additionally, variable and constantly changing levels of antigen expression in different tumor sites further affect CAR-T cell activity. Even worse, TAA are commonly found at low levels on normal tissues, resulting in cross reactions (i.e., “OFF target” and “ON target OFF tumor”) with regional non-tumor cells and severe damage to healthy tissues (Hong et al. 2022).

To date, various methods have been used to overcome these obstacles. Firstly, the most apparent way to tackle tumor TAA heterogeneity and strive for multispecificity is to administer different CAR-T cell products simultaneously or consecutively. It is also possible to combine vectors for two separate CARs during cell production to generate a hybrid product. Another strategy that is rapidly evolving focuses on engineering T-cells with the ability to co-express more than one CARs, namely bispecific or multivalent CARs (Sterner and Sterner 2021). So far there are 3 distinct classes of bispecific CARs that are exploited in T-cell engineering: Dual CAR, tandem CAR (tanCAR) and inhibitory CAR (iCAR). The concept of dual CARs was firstly introduced in 2013, with T-cells expressing both a CAR inducing a suboptimal activation upon recognizing one antigen and an additional chimeric costimulatory receptor specific for a second antigen (Hou et al. 2021). In contrast with dual-T cells that co- express two separate CARs, tanCARs consist of a single receptor that includes two different antigen recognition domains (Marofi et al. 2021). Lastly iCARs, express both a CAR construct and another one that is designed by attaching the signaling domains of T-cell inhibitory receptors to an antigen binder that recognizes a previously specified antigen expressed by healthy cells (Kloss et al. 2013; Hegde et al. 2016). In terms of multivalent CARs, a trivalent CAR-T cell approach has also been tested demonstrating potentiated anti-tumor activity and cytokine secretion over best monospecific and bispecific CAR-T cell designs (Fedorov et al. 2013). Recently, an advanced TRUCKS-Synthetic Notch (synNotch system further upgraded the multivalent approach. SynNotch receptors are a new class of receptors that can induce customized transcriptional circuits in response to recognition of user-specified antigens, connecting antigen sensing to various custom-acquired effector activities (Hashem Boroojerdi et al. 2020). Another strategy to overcome TAA-related is the use of nanobody-based antigen recognition domain instead of an scFv one. Nanobodies in CAR structures are superior to conventional scFv targeting regions, and were additionally used to target specific markers that are overexpressed in TME (Bielamowicz et al. 2018; Roybal et al. 2016; Mo et al. 2021). Lastly a final approach to overcome TAA heterogeneity is to target cancer stem cells, which are one of the reasons for the relapse, metastasis, and broad heterogeneity of tumor cells (Bakhtiari et al. 2009).

### CAR-T cell trafficking and tumor penetration

The complex nature of solid tumors, consisting of numerous tight connections with tumor-surrounding cells, abundant presence of blood vessels, fibroblasts and extracellular matrix proteins, various signaling molecules and decreased levels of oxygen/nutrients, in combination with the surrounding tissues and dense fibrotic matrix in tumor site conform a strong barrier that renders CAR-T cell delivery to tumor site extremely challenging (Xie et al. 2019; Pattabiraman and Weinberg 2014). Additionally, in contrast to hematological malignancies, in which both CAR-T and cancer cells, which share hematopoietic origins, have a higher tendency to migrate to similar locations such as bone morrow or lymph nodes, most solid tumors do not attract CAR-T cells (Pattabiraman and Weinberg 2014; Salmon et al. 2012). That is mainly due to the mismatching of endogenous T-cell chemokine receptors with the chemokines that are secreted from tumor cells. Other crucial elements associated with poor CAR-T cell trafficking and infiltration in solid tumors, are the abnormal secretion of vascular-related factors such as intercellular adhesion molecule 1 (ICAM-1) as well as the presence of other immune cells in tumor tissue. For the latter, recent studies have demonstrated the significance of tissue-resident memory T cells which express a distinctive pattern of adhesion/costimulatory molecules and residency markers (Newick et al. 2016).

The most frequently explored way to potentiate CAR-T cell trafficking and tumor penetration is the administration of CAR-T cells locally in the tumor site. Intracranial and/or intracavitary delivery routes have been assessed exhibiting low toxicity profiles and favorable antitumor activity in patients with glioblastoma (Vitanza et al. 2021; Friedl and Alexander 2011). Similarly, intra-pleural and intra-hepatic artery administrations have been explored in MPM and pancreatibilliary/CRC respectively (Zhan et al. 2019; Hege et al. 2017; Carpenito et al. 2009). It is also possible to exploit implantable biopolymer devices that deliver CAR-T cells directly to the surfaces of solid tumors, thereby exposing them to high concentrations of immune cells for a substantial period of time (Vedvyas et al. 2019). Other engineering approaches are focusing on utilizing chemokines that are excessively secreted by tumor cells by modifying CAR-T cells to express receptors reactive to these chemokines (i.e., C-X-C motif chemokine receptor 8 (CXCR8) in melanoma, C–C motif chemokine receptor (CCR) 2b in neuroblastoma and MPM) (Tang et al. 2021; Smith et al. 2017; Peng et al. 2010). Rather than inducing transgenic expression of chemokine receptors on CAR-T cells, an alternative strategy is to force cancer cells to secrete chemokines in which T-cells are reactive to. Specifically, an oncolytic adenovirus has been exploited to convey chemokine ligand 5 (CCL5) chemokine to the tumor cells. Endogenous T-cells and therefore CAR-T cells typically express RANTES (Regulated upon Activation, Normal T Cell Expressed and Presumably Secreted) receptors (CCR1, CCR3, and CCR5) and the combination of CAR-T cells with the local delivery of CCL5-expressing oncolytic virus has improved persistence of CAR-T cells at tumor sites in preclinical models (Craddock et al. 2010; Kalmpatsa et al. 2020). Beyond addressing the effects of chemokine expression, strategies targeting tumor stroma, dense fibrotic matrix and abnormal vasculature have been examined in preclinical models, utilizing heparinase-secreting CAR-T cells, FAP- specific CAR-T cells and co-administration of anti-VEGF antibodies respectively (Ghosn et al. 2022; Nishio et al. 2014; Guo and Cui 2020).

### Tumor immunosuppressive microenvironment

The immunosuppressive TME is another remarkable hurdle responsible for poor CAR-T cell antitumor activity against solid tumors. A variety of cell types that promote immunosuppression are present in the tumor milieu, including Tregs, myeloid-derived suppressor cells (MDSCs) and tumor-associated macrophages (Caruana et al. 2015). These cells along with tumor cells can also drive tumor growth and proliferation by secreting tumor facilitating cytokines, growth factors and chemokines including but not limited to IL-4, IL-10 and TGF-β (Chinnasamy et al. 2010). Furthermore, immune checkpoint molecules such as PD-1 or cytotoxic T lymphocyte-associated antigen 4 (CTLA-4) and other co-inhibitory pathways can contribute to weak responses to CAR-T cell therapy and promote T cell exhaustion (Quail and Joyce 2013; Binnewies et al. 2018). Lastly, TME is frequently characterized by increased levels of adenosine and reactive oxygen species that are toxic to T cells reducing antitumor responses (Hosseinkhani et al. 2020; Yin et al. 2018).

To date, a number of strategies have been utilized to address the immunosuppressive TME and therefore enhance CAR-T cell therapy effectiveness. The most prominent is the use of combination immunotherapy with CAR-T cells and immune checkpoint inhibitors which target t PD-1/PD-L1 or CTLA-4 inhibitory pathways (Hoskin et al. 2008). CAR-T cells provide the necessary tumor-targeting infiltrate and a highly specific antitumor response while checkpoint blockade therapy can reactivate exhausted immune responses ensuring sustained T cell persistence and function (Hildeman et al. 2003). In solid tumors, this combination therapy is already exploited in several clinical trials (Liu et al. 2020; Carpenito et al. 2009). In the same context**,** PD-1 deficient CAR-T cells (PDCD1 gene knockout) have been generated via CRISPR demonstrating enhanced CAR-T cell antitumor activity in vitro and increased clearance of PD-L1 + tumor xenografts in vivo (Simon et al. 2018). While combination immune checkpoint inhibitors-CAR-T cell therapy will most likely be a new immunotherapy option soon, combining other forms of immunotherapy strategies may still be necessary to fully combat the complex TME (Grosser et al. 2019). Apart from PD-1/PD-L1 or CTLA-4 signaling disruption, many other strategies are currently being tested, tackling different aspects of the hostile TME. In animal models, the depletion of Tregs and/or MDSCs via neutralizing antibodies and genetic modification has augmented CAR-T cell activity (Sterner and Sterner 2021; Rupp et al. 2017). Other engineering approaches have focused on generating CAR-T cells that are resistant to immunosuppression from TGF-β mediated inhibitory signals, through the expression of dominant negative TGF-beta type II receptor (Burga et al. 2015; Zhou et al. 2010) Another appealing strategy involves CAR-T cell manipulation to secrete stimulatory pro- inflammatory cytokines such as IL-12, IL-15 that can modify the TME and potentiate CAR-T cell antitumor responses (Foster et al. 2008; Kloss et al. 2018). Similarly, the already discussed novel TRUCKS-synNotch system can promote the expression of inflammatory cytokines, various antibodies and adjuvants in response to target antigens (Hashem Boroojerdi et al. 2020).

## Conclusion

CAR-T cell therapy has become a promising and effective therapeutic option in patients with hematological malignancies. However, the transition of this technology to solid tumors encounters several challenging biological roadblocks mainly regarding tumor antigen heterogeneity, poor trafficking to tumor site, and hostility of the immunosuppressive TME. Despite an unprecedented number of CAR-T cell clinical trials in solid tumors currently ongoing, all of them are at early stages, and only a limited amount of clinical data has emerged. Therefore, it is of vital importance that more carefully designed clinical trials and multi-center collaborations are performed, and that preclinical research will continue to tackle emerging obstacles via developing elegant solutions and countermeasures, for CAR- T cell therapy to realize its potential as a curative therapeutic approach for solid tumors.

## Data Availability

Data are included in the manuscript.
